# Expanding availability of safe abortion services through private sector accreditation: a case study of the *Yukti Yojana* program in Bihar, India

**DOI:** 10.1186/s12978-015-0096-6

**Published:** 2015-11-10

**Authors:** Sushanta Kumar Banerjee, Kathryn Louise Andersen, Deepa Navin, Garima Mathias

**Affiliations:** Ipas Development Foundation, E-63 Vasant Marg, Vasant Vihar, New Delhi, 110 057 India; Ipas, 300 Market St., Suite 200, Chapel Hill, NC 27516 USA

**Keywords:** Abortion, Accreditation, Evaluation, Health services, India

## Abstract

**Background:**

Recognizing the need to increase access to safe abortion services to reduce maternal mortality and morbidity, the state government of Bihar, India introduced an innovative mechanism of accrediting private health care facilities. The program, *Yukti Yojana* (‘a scheme for solution’), accredits eligible health facilities and supports them in providing abortion-related services free of charge to rural and low-income urban women. This paper describes implementation of *Yukti Yojana*.

**Methods:**

A descriptive analysis of abortion services provided under the Yukti Yojana program was conducted using four data sources: 1) assessment of accredited facilities over 6 months; 2) induced and incomplete abortion service registers; 3) client exit interviews and associated direct observation of client-provider interaction for a sample of accredited facilities; and 4) in-depth interviews with providers and key stakeholders responsible for providing or influencing abortion services. These analyses assessed characteristics of women receiving abortion services, quality of care and client satisfaction, and barriers and facilitating factors of a successful accreditation process.

**Results:**

Forty-nine private facilities were accredited during the first two years of the program, and 84 % had begun providing abortion services, in all 27,724 women were served. Overall, 53 % of beneficiaries reported holding a “Below Poverty Line” card, while 71 % had low living standard. The majority of women (*n* = 569) reported satisfaction (90 %) with their care, while 68 % perceived good quality of services. Having a government-led initiative was considered a key element of success, while stringent requirements for site approval, long waiting time for accreditation, complicated and delayed reimbursement process and low reimbursement fees for abortion services were identified as barriers to implementation.

**Conclusions:**

Yukti Yojana provides a model for successfully involving private OB/GYNs and general physicians to deliver safe abortion services to poor women on a large scale and offers additional evidence that public-private partnerships can be used to ensure availability of high-quality maternal health services to women in low-income countries. Private facility accreditation also offers a promising solution to the limited availability of safe abortion services in low resource settings such as Bihar, India.

## Background

More than half of the 6.4 million abortions performed each year in India are unsafe [1] and almost 5000 abortion-related deaths occur each year in India. This makes unsafe abortion a grave public health issue across India, particularly in the state of Bihar, where the need for safe abortion services is beyond the current capacity of the public health system. Bihar is characterized by poverty and poor reproductive and child health. The state’s maternal mortality ratio (219 deaths per 100,000 births) is considerably higher than the national figure of 178 per 100,000 births [2]. Unsafe abortions continue to be a major contributor to maternal mortality and morbidity in the state.

An estimated 420,000 induced abortions take place in Bihar every year [3], yet data from the state government shows only 704 public sector facilities that are eligible to offer abortion services [4]. Many public sector facilities do not provide safe abortion services because of a shortage of trained and certified providers, which is exacerbated by the frequent practice of transferring trained providers to facilities unequipped for the provision of abortion services.

Given the dearth of abortion services in the public sector, women in Bihar frequently turn to the private sector despite the legal constraints against abortion provision at uncertified private facilities [4]. Only 15 % of the reported abortions in Bihar are conducted in public facilities, whereas 85 % occur in private facilities [4]. This ratio likely underestimates those occurring in the private sector as many cases taking place at unapproved private sites remain uncounted. Because formal private sector safe abortion services often are inaccessible or inadequate, especially to low-income and rural populations, abortions in Bihar often occur outside of government-recognized facilities by untrained providers, possibly under unhygienic conditions.

Private sector abortions also pose a financial burden for women, especially for those requiring hospitalization for post-abortion complications or incomplete abortion as a result of receiving care from unsafe providers or from the use of inappropriate technology [5]. In fact, roughly 24 % of all women hospitalized in a single year fell below the poverty line as a result of the hospitalization and related cost of care^1^ [6], and many women who require emergency obstetric care face significant debt [7].

Private sector facility accreditation for abortion services in Bihar has the potential to increase availability of safe abortion services. Establishing an accreditation process for health facilities can foster sustained facility-level improvements and broader changes at the health-system level. Such accreditation programs typically involve a formal assessment of the degree to which health facilities meet predetermined quality and service availability standards [8, 9]. Accreditation programs may increase equity across health systems and services by ensuring continuous quality improvements [9, 10].

Although accreditation of health care services began in higher-income countries in the early 20th century, there is less information on accreditation in developing countries, particularly for reproductive and child health services [11]. Five countries, Kenya, Zambia, Uganda, Cambodia, and Bangladesh [12–15], did develop accreditation systems but despite substantial improvements in compliance with quality standards, the programs were too resource-intensive to be sustainable in the long term [12]. In Liberia, an accreditation process for health facilities identified the government-led initiative and engagement of stakeholders as key components of success, however the long-term outcomes are not yet known [11].

In India, the concept and practice of accrediting health care facilities is still novel. To ensure the provision of high-quality permanent methods of contraception, the Government of India revised the Quality Assurance Manual in 1996 and has undertaken several new initiatives, including accreditation of health facilities, empanelment of doctors for family planning services, and the introduction of a family planning insurance scheme for both public and private providers [16]. While some states have implemented quality assurance programs (e.g., Gujarat’s private-public partnership designed to provide skilled birth attendance and emergency obstetric care), the most impoverished areas of India lack these programs [17].

Recognizing the need to increase access to safe abortion services to reduce maternal mortality and morbidity, in 2011 the government of Bihar developed a new mechanism for accrediting and subsidizing private health care facilities [18]. The program, *Yukti Yojana* (‘a scheme for solution’), accredits eligible health facilities and supports them in providing abortion-related services free of charge to rural and low-income urban women. This paper describes implementation of *Yukti Yojana*. The findings after 18 months of implementation are promising and may provide a road map for other Indian state governments—as well as other countries—to ensure availability of high-quality, affordable safe abortion services.

## Methods

### Implementation of the Yukti Yojana accreditation program

The *Yukti Yojana* program was implemented under the initiative and leadership of the state government of Bihar, beginning in April 2011 with a press release, newspaper advertisement, and toll-free number for addressing questions of community members and private providers. Communications materials highlighted the program goal of accrediting eligible, private-sector health facilities to provide abortion-related services free of charge. The state health department invited facilities in the private sector to apply for accreditation following standards and guidelines published in both English and Hindi, which detailed the eligibility requirements, application and certification procedures, compensation rates and procedures, and mandatory monitoring of service statistics [18]. To be accredited, a facility had to meet three criteria: (1) at least one gynaecologist or other doctor on site trained to provide abortions, (2) labour or operating rooms, and (3) a system in place for making referrals to a secondary or tertiary level hospital.

A technical advisory group (TAG) of national and local experts on safe abortion service provision was established to monitor the quality and progress of the program. Ipas provided technical support to development of the state’s protocol for accreditation and reimbursement [18], as well as forms used for routine monitoring of services (induced and incomplete abortion registers, 6-monthly facility assessments). A local NGO, GPVS(Gram Praudyogik Vikas Sansthan), was responsible for implementation and monitoring quality of services and reimbursement to accredited facilities. A national level research organization, Center for Media Studies, was subcontracted to conduct independent data collection (CEIs and direct observation) and another organization, Purple Audacity, conducted the IDIs. Finally, Ipas compiled the government service statistics and independently collected CEIs/observation/IDIs to complete an evaluation of the *Yukti Yojana* program.

**Fig. 1:**
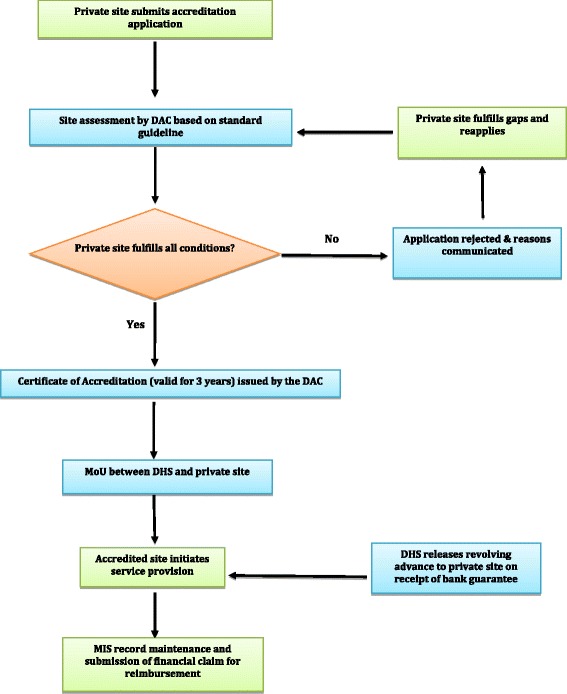
Implementation framework of *YuktiYojana* in Bihar, India

A district accreditation committee (DAC) was established by the state government in each of the 38 districts in Bihar (Fig. 1) to decentralize management of the program. DACs were responsible for promoting the program, approving qualifying facilities, signing memoranda of understanding with accredited facilities, reimbursing facilities for services provided, and monitoring the on-going activities. Facilities were contracted to provide comprehensive abortion care (CAC) services for women seeking care during the first 12 weeks of gestation; treatment, stabilization, or referral for complications from induced or spontaneous abortions; and referral for late abortion. Facilities were reimbursed to cover their costs (medications, consumables, staff salaries and overhead) on a per-case basis based on the type of treatment: induced abortion within 12 weeks of gestation (9.60 USD), abortion-related complications (14.40 USD), and stabilization before making a referral (5.80 USD). Facilities also could be reimbursed for providing a transport subsidy of 3.00 USD to a community-health intermediary for accompanying clients to the accredited facility. In an attempt to encourage early abortion-seeking behavior, facilities were not compensated for performing induced abortions after the first 12 weeks of gestation. This program began as a pilot program, but the government has indicated its intention to continuation, provided sufficient need and resources.

### Monitoring and evaluation

We conducted a descriptive analysis of abortion services provided under the *Yukti Yojana* program using four data sources: 1) assessment of accredited facilities over time; 2) induced and incomplete abortion service registers; 3) client exit interviews and associated direct observation of client-provider interaction for a sample of accredited facilities; and 4) in-depth interviews with providers and key stakeholders responsible for providing or influencing abortion services.

#### Facility assessments

Each facility was assessed immediately after accreditation (baseline) and again at 6 and 12 months post-accreditation using a structured facility assessment tool, which was administered by trained data collectors. Data were captured on facility infrastructure, availability of trained providers, essential drugs, and instrument, client flow, client characteristics, provision of abortion and post-abortion complication services, and quality of service provision. Measures of service provision included use of appropriate technology; provision of post-abortion contraceptives; complete record keeping; availability of site signage and information, education, and communication materials; flow and frequency of reimbursement claims and payments from the district authority; and provider experience with the program.

#### Caseload from MTP and PAC registers

Providers at accredited facilities were also instructed to record individual data on services for induced abortion and post-abortion complications (using a separate evacuation register). Abortion service data included information on client load, client sociodemographic characteristics, types of services provided, post-abortion contraception counselling and method provision, and accompanying outreach workers. Service registers were collected and reviewed periodically, then submitted to the district accreditation committee for reimbursement. Abortion services were judged as including appropriate technologies as recommended by the World Health Organization; these include the use of medical abortion or manual/electric vacuum aspiration.

#### Client exit interviews and observation of client-provider interactions

Sixteen facilities were randomly selected among all accredited facilities using two-stage, stratified random sampling. In the first stage, all accredited facilities were stratified into two geographic regions. In the second stage, eight facilities were selected from each region through systematic random sampling. We recruited all women at least 18 years of age for interview who sought abortion-related services during the two-month period, April to May 2013.

Trained female interviewers used a semi-structured questionnaire to collect data on women’s experiences and perceptions of abortion care at the facility, accessibility of services, attitude and satisfaction with services at the facility, and quality of services received from the facility with regard to appropriate technology, postabortion contraception counselling and service provision, privacy, confidentiality, non-judgmental attitude and respect of providers offering abortion services to women free of cost. To assess the success of the program in targeting low-income women, this study collected socio-economic data, including household durables and BPL (below poverty line) card holding of each woman received abortion services under the scheme. Finally, women were asked to estimate their out of pocket costs for accessing abortion services, including transportation to the health facility, registration cost, doctor’s consultation fee, clinical tests, medicine, general anesthesia and intrauterine contraceptive devices (IUCD) were measured and reported using median and inter-quartile range, as cost data were not normally distributed.

In addition, data collectors observed client-provider interactions using a standard form to assess whether clients were greeted warmly, given the opportunity to discuss medical conditions and help in decision-making, asked about their comprehension of information, encouraged to ask questions, and were addressed by name [11]. Data collectors were stationed in various locations within each facility, including the waiting place, consultation room, recovery room environment, and location for post-abortion contraceptive counselling.

#### In-depth interviews with providers and key stakeholders

We conducted in-depth interviews (IDIs) with 48 providers and stakeholders to capture their experiences with and opinions on: 1) site accreditation program (including its benefits and drawbacks); 2) socio-economic profile of beneficiaries; 3) administrative and management-level barriers experienced by the accredited sites: 4) reimbursement process and flow; and 5) future intentions regarding the program. Participants were purposively selected for the IDIs to ensure a range of types of providers, including general physicians (*n* = 9), specialized Ob/Gyns (*n* = 11), nursing staff (*n* = 10), district program manager and NGO workers (*n* = 8), and community outreach workers (*n* = 5). Four trained senior researchers conducted the IDIs, which were audio recorded with prior consent of the respondents.

The project was reviewed and approved by the Technical Advisory Group (TAG), and Institutional Review Boards (IRB) in India and USA, respectively. Informed consent was obtained by all clients, providers and stakeholders before participation in interviews.

### Measures

A client satisfaction index was computed based on nine parameters measured in the client exit interview, including 1) overall client satisfaction (not at all, somewhat dissatisfied, neutral, somewhat satisfied, or completely satisfied); 2) client’s rating of this facility compared to other facilities that provide sexual and reproductive health services (much worse, somewhat worse, same, somewhat better, or much better); 3) future intention to come back to this facility (no/yes); 4) intention to recommend this facility to others (no/yes); 5) sufficient time given by the doctor (no/yes); 6) non-judgmental behavior by staff at the facility (no/yes); 7) non-judgmental attitude of the staff (yes/no); 8) ability to express client’s individual concerns and questions (no/yes); and 9) client’s perception of whether free service affected care from the provider (no/yes). The client satisfaction index was computed by summing ordered scores for each item and ranged from 0 to 14. The score was then categorized programmatically as high, moderate, and low based on composite scores 13–14, 10–12 and <10 respectively.

Similarly, a quality of care index was computed based on seven parameters reported by clients or observed by the study observers, including: 1) information on all methods of abortion available at site (observation); 2) client or husband/relative involvement in deciding abortion method (self-report); 3) audio and visual privacy reported by client; 4) observed privacy of the client-provider interaction; 5) counselling on post abortion contraception (self-report); 6) acceptance of a contraceptive method immediately following the procedure (self-report); and 7) information provided on when follow-up might be needed (self-report). The composite score for quality of care was computed by summing the number of parameters present and ranged from 0 to 7. The composite quality of care scores were then characterized as high, moderate, and low based on composite scores 6–7, 4–5, and <4 respectively.

Mean facility caseload was computed and graphed by month of the project. A linear regression line was fit to the data to investigate whether mean caseload per month was increasing, after adjusting for the observed cyclic variation using a 3-month moving average. The slope and R^2^ from the fitted regression line are reported. All quantitative analysis was conducted using SPSS 13.0.

### Data analysis

Facility monitoring data and service statistics are described using frequencies and percentages for categorical data or means and standard deviations for continuous, normally distributed data. Skewed data is presented as medians and range (minimum, maximum).

In-depth interviews were transcribed and coded independently by two researchers using Atlas.ti 7. Inter-coder agreement checks were conducted, with adjustments to the codebook and recoding of text as needed. Codes were cluster-analyzed, most notably to understand barriers to implementation of the *Yukti Yojana* program.

## Results

**Fig. 2:**
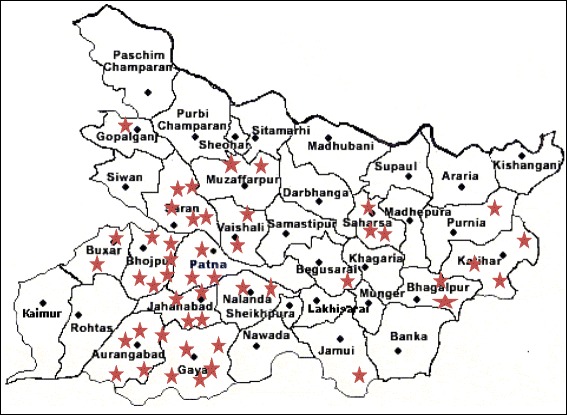
Location of 49 private sites accredited under *YuktiYojana* program as of May 2013 (stars indicate accredited health facilities)

Between July 2011 and December 2012, 69 private health facilities applied for accreditation, with 49 receiving accreditation under the *Yukti Yojana* program by May 2013. Newly accredited private facilities are located in 18 of Bihar’s 38 districts at the time of implementation (Fig. 2).

The accreditation process was effective in both clinics and nursing home/private hospitals (Table 1). The overwhelming majority of facilities (94 %) obtained information about the accrediting process from the implementing NGO. The mean time between submitting application and DAC’s inspection was 3.7 months; while the mean time between DAC’s inspection and securing the DAC’s approval was 1.5 months.

**Table 1:** Accreditation process for 49 private facilities accredited for safe abortion services in Bihar, India under *Yukti Yojana*

	Number	Percent
Process characteristics		
Facility type		
Clinic	26	(53)
Nursing home/private hospital	23	(47)
Source of information regarding accreditation program^a^		
Newspaper advertisement or government website	5	(10)
Implementing NGO	46	(94)
Other	1	(2)
Time from application to DAC inspection		
≤ 1 month	8	(16)
2–3 months	18	(37)
> 3 months	23	(47)
Mean time to inspection, in months		
Mean	3.7	
(sd)	(3.46)	
Time from DAC inspection to approval		
≤ 1 month	33	(67)
2–3 months	9	(18)
> 3 months	7	(14)
Mean time to inspection, in months		
Mean	1.5	
(sd)	(0.74)	

*NGO* non-governmental organization, *DAC* District Accreditation Committee, *SD* standard deviation

^a^Percentages may sum to more than 100 % because multiple responses were possible

### Facility assessments

Immediately after DAC approval, baseline facility data was collected from the 49 approved sites (Table 2), this assessment was repeated at 6, 12 and 18 months post-accreditation. However, as sites were accredited at different points of time during the first 18 months of the project, not all 49 sites are eligible or have received, all follow-up assessments. At baseline, 98 % of facilities had one or more MTP-trained doctors providing abortion services. Facilities served a mean of 82 women per day for all sexual and reproductive health care services, including abortion, postabortion complications and other reproductive health issues. Although all facilities (100 %) provided abortion care in the 3 months preceding the baseline survey, only 74 % had all essential equipment and drugs.^2^ Over time, most indicators were stable or improved slightly, with the exception of maintaining referral linkages.

**Table 2:** Findings of baseline, 6-Month, 12-Month, and 18-Month assessments among 49 Facilities in Bihar accredited for abortion services under *Yukti Yojana*

	Baseline (*n* = 49)		6-Month (*n* = 45)		12-Month (*n* = 36)		18-Month (*n* = 32)	
	*n*	(%)	*n*	(%)	*n*	(%)	*n*	(%)
Facility infrastructure								
Having at least one MTP-trained doctor	48	(98)	44	(98)	35	(97)	31	(97)
Necessary instruments and supplies for infection control	48	(98)	45	(100)	36	(100)	32	(100)
Displayed IEC materials on MTP	41	(84)	45	(100)	36	(100)	32	(100)
*Yukti Yojana* site signs displayed	41	(84)	45	(100)	36	(100)	32	(100)
Poster displayed noting that MTP services are free	41	(84)	45	(100)	36	(100)	32	(100)
Maintain separate MTP logbook/register	46	(94)	45	(100)	36	(100)	32	(100)
All essential equipment and drugs for MTP	36	(74)	43	(96)	36	(100)	29	(91)
Service provision								
Number of adult women coming for SRH facilities per day								
Mean	82		81		85		85	
(SD)	(20.15)		(23.52)		(21.20)		(19.11)	
Provided MTP services in the past 3 months	45	(92)	45	(100)	35	(97)	31	(97)
Providing postabortion contraceptives	43	(96)	45	(100)	35	(97)	31	(97)
Providing MTP services 7 days/week	33	(67)	39	(87)	28	(78)	26	(81)
Referral linkages for second trimester and serious complications	40	(82)	36	(80)	26	(72)	25	(78)
Treatment area has audio and visual privacy	49	(100)	45	(100)	36	(100)	32	(100)

*MTP* medical termination of pregnancy, *IEC* information, education and communication, *SRH* sexual and reproductive health, *SD* standard deviation

**Fig. 3:**
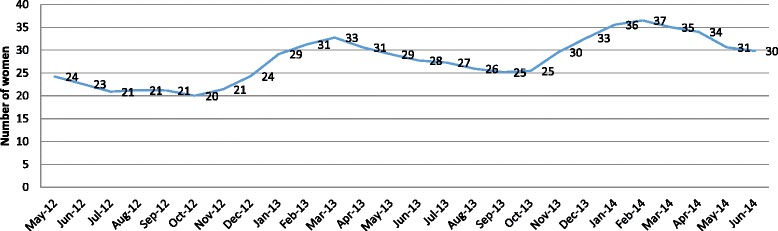
Three-monthly moving average of abortion caseload per month per site at *Yukti Yojana* accredited facilities between May 2012 and July 2014

By June 2014, 8 (16 %) facilities had yet to provide abortion services under the *Yukti Yojana* program, despite accreditation. The primary barriers to service provision were administrative—for example, six sites willing to provide services were not able to do so because of the state government regulation that private sites with a part time or visiting provider from the public sector were not eligible for accreditation. Two additional sites had non-functional operating theaters. The 41 accredited facilities provided abortion care services to 27,724 women between January 2012 and July 2014 (Table 3), 46 % were induced. Just 104 women (<1 %) were referred to higher level facilities for second trimester or severe complications. Abortion clients had a mean age of 29.9 years (SD = 4.2 years), the majority were Hindu (92 %) and almost a quarter (24 %) had never attended school. Almost all women (98 %) presented for care within the first 12 weeks of gestation. Among those who received abortion care, approximately 93 % received uterine evacuation services with appropriate technologies including manual/electric vacuum aspirator or medical abortion. More than four-fifths (93 %) of women received contraception immediately after the procedure. As shown in Fig. 3, mean caseload per facility per month increased slowly over time, with some seasonal variation. The seasonal variation in caseload was observed at the beginning of the new financial year (April 2013 and April 2014). As depicted in Fig. 3, mean caseload started showing declining trend from March 2013 and continued till October 2013. A similar trend was observed in the next financial year (April 2014 onwards), likely due to the irregular flow of reimbursement.

**Table 3:** Description of clients and abortion care for 27,724 abortion cases at 41 facilities in Bihar accredited under *Yukti Yojana,* January 2012 to July 2014

	All MTP cases (*N* = 27,724)	
	*n*	(%)
Type of service received		
Induced abortion	12,627	(46)
Postabortion care	14,993	(54)
Referred to higher level facility	104	(<1)
Missing	0	
Client age, years^a^		
17–24	2381	(16)
≥ 25	12,265	(82)
Missing	347	(2)
Age		
Mean	29.9	
(SD)	(11.42)	
Client religion^a^		
Hindu	13,723	(92)
Muslim	1182	(8)
Other	11	(<1)
Missing	77	(1)
Client education		
Never attended school	6624	(24)
Primary/middle school	14,018	(51)
Secondary school or more	5253	(19)
Missing	1829	(7)
Client caste		
SC/ST	4434	(16)
OBC	14,128	(51)
General	8155	(29)
Missing	1007	(4)
Duration of pregnancy at presentation		
≤ 8 weeks	16,689	(60)
9–10 weeks	7276	(26)
11–12 weeks	3306	(12)
> 12 weeks	163	(1)
Missing	290	(1)
Mean	8.4	
(SD)	(1.96)	
Appropriate technology for uterine evacuation^b^		
Yes	25,821	(93)
No	545	(2)
Missing	1254	(5)
Received postabortion contraception^b^		
Yes	25,635	(93)
No	744	(3)
Missing	1241	(4)

*SC* scheduled caste, *ST* scheduled tribe, *OBC* other backward classes

^a^Percentage computed among the 14,993 induced abortion cases because client age and religion were not collected via the Incomplete Abortion Case Register

^b^Percentage computed among the 27,620 cases not referred to a higher-level facility

### Client exit interviews

Client exit interviews were conducted with 569 women at the randomly selected subset of accredited facilities. As shown in Table 4, one-fourth of women were less than 25 years and the overwhelming majority of women who received abortion services were married (98 %). More than one-third of women (37 %) had never attended school, while another one-third had primary or middle level of schooling. According to the Government of India’s caste designations, most were scheduled caste or scheduled tribe (19 %) or other backward caste (65 %). More than half of women reported holding a BPL (below poverty line) card, while composite SLI (standard of living) index derived through possession of consumer durables revealed poor economic status for the majority of the beneficiaries (71 %). Most women came from nearby villages (66 %) and towns (8 %), traveling an average distance of 16.6 km. More than half of women (58 %) decided to come to these accredited private facilities mainly because abortion services were available free of cost (data not shown). Although abortion services were free for all women, 96 % of respondents incurred average (Median) out of pocket costs of 3 USD, ranging from 0 to 52 USD, primarily for transportation and food, registration fee at the facility and other medicines. Service quality was rated equally across the index as 33 % high quality, moderate for 36 % and low quality for 31 %. Women were also asked to express their satisfaction levels in terms of providers’ non-judgmental attitudes and behaviors. The client satisfaction index showed that slightly less than half the women coming for abortion services (46 %) expressed very high levels of satisfaction compared to 44 and 10 % who expressed moderate or low level of satisfaction, respectively.

**Table 4:** Sociodemographic profile, client satisfaction index and quality of services index among 569 women who received abortion services at 16 selected accredited facilities between April and May 2013

	Number	Percent
Type of service received		
Induced abortion	325	(57)
Postabortion care	144	(43)
Marital status		
Married	558	(98)
Not married	11	(2)
Living children		
None	40	(7)
1–2	245	(44)
≥ 3	287	(49)
Holding BPL card		
Yes	303	(53)
No	266	(47)
Wealth index		
Low	404	(71)
Medium	160	(28)
High	5	(1)
Place of residence		
Same town	151	(26)
Other town	44	(8)
Village	374	(66)
Distance traveled in km		
Mean	16.6	
(SD)	(17.3)	
Client satisfaction index^a^		
High	261	(46)
Moderate	249	(44)
Low	59	(10)
Quality of services index^b^		
High	185	(33)
Moderate	205	(36)
Low	179	(31)

*BPL* below poverty line

^a^Client Satisfaction Index, range 0–14: Low = 0–9, Moderate = 10–12, High = 13–14

^b^Quality of Services Index, range 0–7: Low = 0–3, Moderate = 4–5, High = 6–7

### In-depth interviews

In-depth interviews with 48 respondents from the public and private sectors were used to help identify the operational bottlenecks experienced as part of the *Yukti Yojana* program. The most frequently identified problems included stringent requirements for site approval, long waiting time for site approval, complicated and delayed reimbursement process, and low reimbursement fees for services. As expressed by a few of the respondents:

*‘…After submitting application we followed up with district authority twice and … again after DAC inspection we did the same. It is a time taking and complicated procedure’ [Provider; NGO/Trust hospital]*

*‘The existing fees at private sector is substantially higher than the rate assigned under this scheme. Sometime we feel very difficult to manage our own internal cost’*

*[Ob/Gyn provider; Private Clinic]*

*‘Reimbursement process takes its own time. Government is managing 100 things at time… We always need to follow-up for our due payment. This is demotivating.’*

*[Ob/Gyn Provider; Private clinic]*

## Discussion

This analysis suggests that *Yukti Yojana* program developed by the state government of Bihar is the first successful accreditation program for provision of safe abortion services in private sector facilities without charging service fees to women.

In the first 18 months of *Yukti Yojana*, 49 private facilities successfully completed the accreditation process. Facilities were accredited relatively slowly, with an average duration of just over 5 months between application and approval. Widespread interest on the part of facilities in becoming accredited likely reflects the crucial role that the private sector holds in addressing women’s reproductive health needs as well as the recognition of the importance of subsidizing services to low-income women. The experience in Bihar is in line with a global movement experimenting toward public-private models of ensuring the poorest women have access to reproductive health care, including accreditation and subsidies through vouchers to private and public facilities being undertaken in Liberia, Kenya and Bangladesh [11, 13, 15]. Although the wait time between application and approval has been long due to administrative protocols, abortion services were established at facilities quickly upon accreditation.

Almost all clients (86 %) presenting for abortion care were seen within the first 10 weeks of gestation (mean 8.4 weeks), suggesting that removing financial and other barriers that women face when accessing induced abortion services may result in presentation for abortion services at earlier gestational age, which can result in fewer complications for women and lower costs for the health care system. Improvements in facility infrastructure and service provision ensured that these women would have routine access to high-quality services. Over the course of the project, availability of all essential equipment and drugs for MTP improved from 74 % (of facilities reporting availability) at baseline to 100 and 91 % at 12- and 18-month follow-up, respectively. Furthermore, at baseline only 67 % of facilities provided MTP seven days a week compared to 81 % at 18-month follow-up.

Although concerns have been raised that such private accreditation scheme may not target rural and poor women, the findings of the client-exit-interviews showed that more than half of *Yukti Yojana* program beneficiaries (53 %) had a BPL card (below poverty line identity card assigned by the local government based on their household income) and two-third of beneficiaries were from rural areas. Furthermore, the composite wealth index indicated that 71 % of beneficiaries held low standard of living, indicating that the scheme adequately targeted poor women. Low levels of education and caste structure also suggest utilization of services by poor and vulnarable women.

Furthermore, the *Yukti Yojana* program helped women access free *high quality* abortion services in the private sector. Monitoring data of accredited sites suggests that abortion service quality is in line with data from the public sector in Bihar for 38,879 women served between July 2011 and June 2014 [19] and much improved over 24,973 women served by private sector unaccredited facilities between February and May 2011 [4]. While only 46 % of unaccredited private sector abortion clients were served with appropriate uterine evacuation technology [4] 93 % of *Yukti Yojana* clients and 98 % of public sector clients were served with appropriate technology; similarly, 89 % of private sector abortion clients received postabortion contraception as compared to 93 % of *Yukti Yojana* clients and 94 % of public sector abortion clients. Furthermore, uptake of postabortion contraception immediately after the procedure was a major contributor to the high perceived client satisfaction and quality of care indices.

Although abortion services were offered free of cost, women reported some out of pocket cost to meet other expenses including transport, medicine, clinical tests, food and site registration fee. However, these out of pocket costs were low compare to other studies. For example, a study conducted in Jharkhand [20] in almost a similar situation where abortion services were free, the average out of pocket cost (USD 10) was significantly higher than the cost incurred by *Yukti Yojana* clients.

As was seen in Liberia [11], the success of *Yukti Yojana* has been driven by government ownership of the initiative and strong engagement from stakeholders for implementation and monitoring. However, providers and stakeholders raised major concerns on the bureaucratic complexity of approval process and fund reimbursement. These two factors have also been perceived as important attributes of program sustainability in Bihar, as well as in other settings. After initial review of the analysis, the state government of Bihar increased the reimbursement fees to ensure sustainability of the program. Longer-term monitoring will provide more information on their success addressing this barrier. In addition, before expansion of *Yukti Yojana* to all 38 districts of Bihar, it will be important to continue efforts to expand access to safe abortion within the public health system as well. This approach will not only increase availability of services, but will improve equity in access.

This study has several limitations. Ideally, the study design would have incorporated non-accredited comparison sites through a quasi-experimental design. In addition, observation of provider-client interaction was not possible, which would have provided more information about the quality of services according to existing standards and guidelines. Finally, longer-term impact on service availability and sustainability will not be known until the next round of accreditation and facility assessment in mid-2016.

Despite limitations in causation, the program made notable advances on a number of levels. A key contribution was in creating an accreditation model with a sustainable approach. Often times, accreditation schemes become defunct due to lack of administrative capacity or budgetary constraints. In *Yukti Yojana* the state government successfully implemented the program for two full years, and created a detailed hand-over and scale-up plan, which included developing accreditation protocols and site evaluation forms that could be replicated by other states. Beyond its sustainability, the program was shown to be successful in serving poor women, who are most at-risk for unsafe abortion and related morbidity and mortality [21]. These women were reached specifically through provision of free services, which was a critical element of the *Yukti Yojana* design. Free services in and of themselves are desirable, but client exit interviews further demonstrated the high quality of care through satisfaction rates. Through its design and implementation, the *Yukti Yojana* project was successful in reaching women most at-need for safe and quality abortion services.

### Conclusions

*Yukti Yojana* provides a model for successfully involving private health care facilities to deliver safe abortion services and care to poor women on a large scale and offers additional evidence that public-private partnerships can be used to ensure availability of high-quality maternal health services to women in low-income countries.

## Abbreviations

BPL: Below poverty line
CAC: Comprehensive abortion care
CEI: Client exit interview
DAC: District accreditation committee
IDI: In-depth interview
IEC: Information, education and communication
IRB: Institutional review board
IUCD: Intrauterine contraceptive device
MTP: Medical termination of pregnancy
NGO: Non-governmental organization
Ob/Gyn: Obstetrician/Gynecologist
PPP: Public-private partnership
SD: Standard deviation
SLI: Standard of living index
TAG: Technical advisory group
USD: United States dollars
WHO: World Health Organization

## Footnotes

1 The movement of patients into poverty was determined by the proportion of hospital patients whose assets would fall below poverty line after direct hospitalization costs were deducted from their existing measured resources. The definition of “poverty line” was not provided in this article.

2 Equipment essential for induced abortion services include OT or labour table, suction machine for MTP (MVA or EVA), speculum, valsellum or tenaculum, suction cannulae, blunt and sharp curette, cheattle’s forceps, dialator set, kidney tray, autoclave, Boiler and steam sterilizer, sponge holder, chlorine solution, antiseptic solution, atropine injection, Ampicilin, Amoxicillin, or Trihydrate, or Cephalexin or a suitable alternative, Paracetamol, Penatazocine, Dicyclomine or a suitable alternative.
